# Obesity and depression: Review on common neurobiological mechanisms and identification of potential drug targets.

**DOI:** 10.1192/j.eurpsy.2024.1106

**Published:** 2024-08-27

**Authors:** J. R. Lopez, R. Zaghi-Lara

**Affiliations:** ^1^Posgrado de psiquiatría, Universidad de San Carlos de Guatemala; ^2^President, Asociación Psiquiaátrica de Guatemala; ^3^CIGua, Comunidad de Investigación de Guatemala, Guatemala, Guatemala

## Abstract

**Introduction:**

This review aimed to identify common pathophysiological mechanisms that exist between depression and obesity, as well as pharmacological strategies used in clinical trials and animal models. It is necessary to carry out larger studies that integrate the multiple neurobiological processes of these phenomena and the search for therapeutic targets that affect these pathways.

**Objectives:**

Conduct a literature review on the common neurobiological aspects that exist between depression and obesity. Compare pharmacological and therapeutic strategies in the management of depressive patients by means of common neurobiological mechanisms.

**Methods:**

We used the Pubmed search engine to search for the keywords: ((depression and obesity) OR (common pathways for depression and obesity) OR (therapeutic targets for depression and obesity) OR (neurobiology of depression and obesity) OR (treatments for depression and obesity))Once the predefined screening was carried out, 68 studies were identified. 54 articles were left for review and analysis. At the end of the review, 25 studies were discarded, including 29 studies with relevance to the objectives described in the study. These articles were selected when they provided information with adequate, concrete and specific reasoning towards the scientific and methodological elements of the review.

**Results:**

**
Table 1:** Findings related to the NEGR1 gene and its involvement in processes associated with alterations associated with depression and obesity.

**
Table 2:** Findings of inflammation associated with depression and obesity in clinical trials and animal models.

**Image:**

**Figure:**
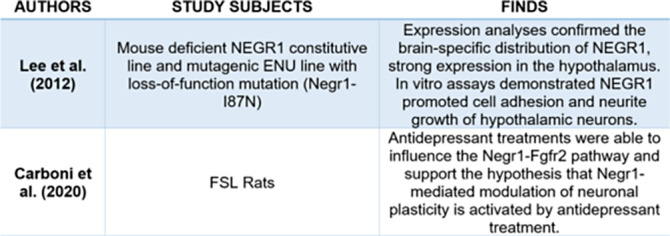

**Image 2:**

**Figure:**
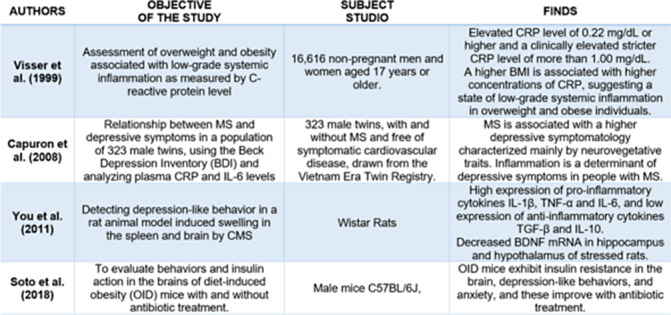

**Conclusions:**

Alterations in the NEGR1 gene, inflammatory markers, HPA axis and microbiota demonstrate multiple pathophysiological mechanisms in the clinical pictures associated with obesity and depression.

Infliximab, pioglitazone, ondansentron, BVT.2733 and palmitoylethanolamide showed anti-inflammatory regulatory effects with reduction in depressive symptoms and multiple anti-inflammatory markers.

Animal models for obesity and depression present ample and reliable evidence regarding the use of drugs that direct their therapeutic profile towards the pathophysiological mechanisms involved in pathologies involving depressive and metabolic disorders.

**Disclosure of Interest:**

None Declared

